# Falls risk is predictive of dysphagia in Parkinson’s disease

**DOI:** 10.1007/s10072-021-05700-6

**Published:** 2021-11-03

**Authors:** Christopher Kobylecki, Irena Shiderova, Mihaela Boca, Emilia Michou

**Affiliations:** 1 Department of Neurology, Salford Royal NHS Foundation Trust, Manchester Academic Health Science Centre, University of Manchester, Manchester, UK; 2 Department of Neurology, Manchester Centre for Clinical Neurosciences, Salford Royal NHS Foundation Trust, Stott Lane, Salford, M6 8HD UK; 3 School of Medical Sciences, Faculty of Biology, Medicine and Health, University of Manchester, Manchester, UK; 4 Department of Neurology, Bristol Brain Centre, North Bristol NHS Trust, Bristol, UK; 5 Division of Diabetes, Endocrinology and Gastroenterology, School of Medical Sciences, Faculty of Biology, Medicine and Health, University of Manchester, Manchester, UK; 6 Department of Speech and Language Therapy, School of Health Rehabilitation Sciences, University of Patras, Patras, Greece

**Keywords:** Parkinson’s disease, Falls, Dysphagia

## Abstract

**Objective:**

Evaluate the relationship between falls, freezing of gait, and swallowing disturbance in Parkinson’s disease (PD).

**Background:**

Dysphagia is a common symptom in PD, and is often thought of as an axial feature along with falls and gait disturbance. It is of interest to examine the relationship between these symptoms in PD, given the possibility of shared pathophysiology due to non-dopaminergic and extranigral dysfunction.

**Methods:**

We recruited 29 consecutive non-demented patients with idiopathic PD and at least one clinically determined impairment in swallowing, falls, or freezing of gait. Swallow dysfunction was assessed using the Swallowing Disturbance Questionnaire (SDQ). The Falls Efficacy Scale and Freezing-of-gait questionnaire were recorded. Correlation analysis and multiple regression were used to determine the relationship between swallow and gait disturbance.

**Results:**

Total SDQ score correlated strongly with the falls efficacy scale (Spearman’s rho = 0.594; *P* = 0.001), but not with the freezing-of-gait score. Linear regression controlling for other factors associated with dysphagia identified falls efficacy score as a significant predictor of swallow dysfunction.

**Conclusions:**

The severity of dysphagia in PD is closely related to severity of falls, but not gait freezing. This may be helpful to more precisely determine the anatomical substrate of levodopa-resistant axial symptoms in PD and provide clues to further management.

## Background

Dysphagia is a common feature of Parkinson’s disease (PD) and is implicated in potential adverse health outcomes such as aspiration pneumonia and hospitalization. Factors associated with dysphagia in PD include age, disease duration, and cognitive impairment [1]. However, while often considered as a feature of late-stage PD, swallowing impairments have been described earlier in the disease course, while the method of detecting dysphagia may influence the prevalence [2]. Dysphagia is often classed as a levodopa-resistant “axial” PD symptom, similar to gait problems including falls and freezing of gait (FOG) [3]. However, the relationship between dysphagia and axial gait disturbance in PD has not been assessed in detail.

We hypothesized that falls risk and freezing of gait would be associated with dysphagia in people with PD. Our aim was to recruit a sample of patients with gait disturbance and/or dysphagia to explore the relationship between these symptoms.

## Methods

We performed a questionnaire-based study in 29 consecutive non-demented outpatients with PD who reported at least one symptom of swallow impairment, postural instability, or FOG. The study was approved by London Chelsea Research Ethics Committee (reference 17/LO/0384) and all participants gave written informed consent. We recorded demographic information and data on PD duration and medication doses; levodopa equivalent daily dose (LEDD) was calculated as previously described [4]. Dysphagia was assessed using the Swallow Disturbance Questionnaire [5], validated in PD to assess oral and pharyngeal components of swallow. Fear of falling was assessed with the Falls Efficacy Scale [6], while FOG was measured using the Freezing of Gait Questionnaire [7]. Statistical analysis was performed using SPSS 23.0 (IBM).

## Results

**Fig. 1:**
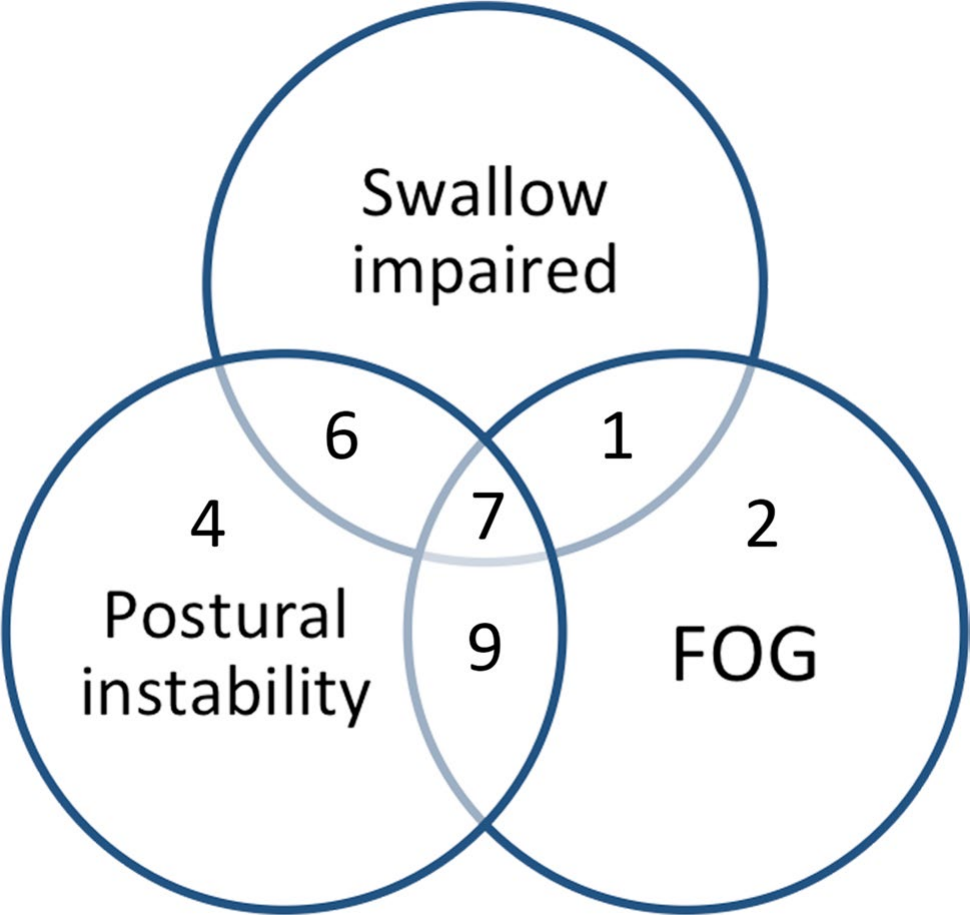
Diagram illustrating proportion of participants with different core symptoms of swallow impairment, postural stability, or freezing of gait (FOG)

The demographic characteristics of the study population and their questionnaire scores are detailed in Table 1. Postural instability was reported by 26 patients, whereas swallowing problems were reported by 14 and FOG by 19; 23 patients reported impairment in more than one domain (Fig. 1).

**Table 1:** Demographic characteristics and study outcomes. *LEDD*, levodopa equivalent daily dose; *SDQ*, Swallow Disturbance Questionnaire. Data are presented as mean (standard deviation) aside from those indicated with * which are median (interquartile range)

Variable	Result
Gender	Male 11 Female 18
Age (y)	70 (7.7)
Disease duration (y)	12.1 (5.7)
LEDD (mg)	975 (429)
SDQ oral*	3.0 (1.0–5.0)
SDQ pharyngeal*	4.5 (2.0–9.5)
SDQ total*	8.5 (2.0–14.5)
Falls efficacy scale total	46.8 (21.6)
Freezing of gait score	13.6 (5.2)

The FES total score correlated positively with SDQ total score (Spearman’s rho = 0.594; *P* = 0.001), as well as oral (rho = 0.619; *P* < 0.001) and pharyngeal subscales (rho = 0.563; *P* = 0.001). In contrast, neither SDQ total (rho = 0.176; *P* = 0.362) nor subscales showed a correlation with FOG Questionnaire score.

We performed multiple linear regression with total SDQ as the dependent variable using age, gender, disease duration, LEDD, and FES total score as predictive variables. In this model, FES total score was a significant predictor of SDQ score (standardized *β* = 0.533, *P* = 0.004), whereas other variables did not reach statistical significance.

## Discussion

Our study shows that fear of falling, an indirect measure of falls risk, but not FOG is associated with dysphagia in a population of moderate-advanced PD without dementia. In addition, falls risk was predictive of dysphagia severity even when other factors previously linked with swallowing dysfunction were taken into account. These data suggest a link between these two axial PD symptoms beyond general disease progression. Walker and colleagues previously identified a correlation between dysphagia and falls identified by the Unified Parkinson’s Disease Rating scale part II, but did not use other validated assessment tools for these symptoms [8].

The causes of falls and gait disturbance in PD are multifactorial, but accumulating evidence points to dysfunction of the cholinergic pedunculopontine nucleus (PPN) as a key factor. Oropharyngeal swallowing is mainly controlled by the nucleus ambiguus, which is spared in PD but receives modulatory input from suprabulbar pattern generators including PPN [9]. We propose that degenerative changes in PPN could therefore provide a common substrate for parallel gait and swallow dysfunction in PD. However, further mechanistic work would be required to confirm this hypothesis.

The limitations of our work include a relatively small sample size but otherwise enriched for the presence of clinical features of interest. Despite having used a validated questionnaire for dysphagia in PD which has shown good sensitivity and specificity [5], we acknowledge that imaging techniques such as fiberoptic endoscopic evaluation of swallowing (FEES) can potentially verify swallowing problems. While gait was not assessed objectively, both FES and FOG questionnaire are recommended by current guidance in PD [10].

Our data suggest a specific relationship between falls and dysphagia in PD. The development of falls should alert clinicians to the possibility of other axial features like dysphagia. Further prospective work in a larger population and mechanistic studies of the role of brain areas such as PPN would help clarify this relationship.

## Data Availability

Data will be provided upon reasonable request.
